# Do reactive oxygen species damage or protect the heart in ischemia and reperfusion? Analysis on experimental and clinical data

**DOI:** 10.7555/JBR.36.20220261

**Published:** 2023-07-28

**Authors:** Leonid N. Maslov, Natalia V. Naryzhnaya, Maria Sirotina, Alexandr V. Mukhomedzyanov, Boris K. Kurbatov, Alla A. Boshchenko, Huijie Ma, Yi Zhang, Feng Fu, Jianming Pei, Viacheslav N. Azev, Vladimir A. Pereverzev

**Affiliations:** 1 Cardiology Research Institute, Tomsk National Research Medical Center, the Russian Academy of Sciences, Tomsk, Tomsk Region 634012, Russia; 2 Department of Physiology, Hebei Medical University, Shijiazhuang, Hebei 050017, China; 3 Department of Physiology and Pathophysiology, National Key Discipline of Cell Biology, School of Basic Medicine, the Fourth Military Medical University, Xi'an, Shaanxi 710032, China; 4 The Branch of the Institute of Bioorganic Chemistry, Russian Academy of Sciences, Pushchino, Moscow Oblast 142290, Russia; 5 Department of Normal Physiology, Belarusian State Medical University, Minsk 220083, Belarus

**Keywords:** reactive oxygen species, free radicals, antioxidants, heart, reperfusion, patients

## Abstract

The role of reactive oxygen species (ROS) in ischemic and reperfusion (I/R) injury of the heart has been discussed for more than 40 years. It has been demonstrated that reperfusion triggers a multiple increase in free radical generation in the isolated heart. Antioxidants were found to have the ability to mitigate I/R injury of the heart. However, it is unclear whether their cardioprotective effect truly depends on the decrease of ROS levels in myocardial tissues. Since high doses and high concentrations of antioxidants were experimentally used, it is highly likely that the cardioprotective effect of antioxidants depends on their interaction not only with free radicals but also with other molecules. It has been demonstrated that the antioxidant N-2-mercaptopropionyl glycine or NDPH oxidase knockout abolished the cardioprotective effect of ischemic preconditioning. Consequently, there is evidence that ROS protect the heart against the I/R injury.

## Introduction

In the 1980s, it was widely believed that reactive oxygen species (ROS) played an important and perhaps even a key role in ischemic/reperfusion (I/R) injury of the heart^[1–2]^. Attempts have been made to create cardioprotective drugs based on the antioxidant ionol (butylated hydroxytoluene, BHT) for treatment of acute myocardial infarction (AMI)^[3]^. However, in the 21^st^ century, an opinion has formed that ROS can exhibit cardioprotective properties and increase cardiac tolerance to I/R^[4–5]^. However, even today, many investigators still hold the opinion that ROS are involved in I/R injury of the heart^[6–9]^.

In this review, we attempted to analyze the data in favor of either a negative or protective role of ROS in I/R injury of the heart.

## Types of ROS and their external and myocardial origins

### Types of ROS

The following species of ROS are generated in humans and animals: superoxide radical (O_2_^•−^), hydroxyl radical (^•^OH), singlet oxygen (^1^O_2_), hydrogen peroxide (H_2_O_2_), peroxyl radical (PLOO^•^), and fatty acid hydroperoxides (LOOH)^[5,10–14]^. It was hypothesized that ^•^OH was formed in the Fenton reaction^[5,10–11,13]^.

1 $
\mathrm{Fe}^{2+}+\mathrm{H}_2 \mathrm{O}_2 \rightarrow \mathrm{Fe}^{3+}+\mathrm{OH}^{-}+{} \cdot \mathrm{OH}$

### Extracellular sources of ROS

Myocardial reperfusion induced leukocyte invasion in the area at risk^[15]^. Neutrophils and macrophages contain NADPH oxidase (NOX), which produces O_2_^•−[15]^. Neutrophils express the enzyme myeloperoxidase, which synthesizes hypochlorous acid (HOCl) and other oxidants (HOSCN and HOBr)^[16]^. These oxidants can kill microorganisms but also injure mammalian cells^[16]^. HOCl could be involved in the synthesis of singlet oxygen^[17]^.

2 $
\mathrm{HOCl}+\mathrm{H}_2 \mathrm{O}_2 \rightarrow \mathrm{HCl}+{ }^1 \mathrm{O}_2+\mathrm{H}_2 \mathrm{O}$

### Intracellular sources of ROS

It has been reported that mitochondria are the main source of ROS in cardiomyocytes^[13]^. Superoxide radical is generated in mitochondria by complex Ⅰ and complex Ⅲ. The O_2_^•−^ is transformed into H_2_O_2_^[13]^. NADPH oxidase is another important source of superoxide synthesis in cardiac mitochondria^[5,13]^. Superoxide radical is transformed into H_2_O_2_ spontaneously or by superoxide dismutase (SOD)^[5]^. The xanthine oxidase (XO) is also an important source of O_2_^•−^ in rats, but not in humans or rabbits^[5]^. Uncoupled NO-synthase (NOS) appears in cells in the setting of hypoxia^[5,13]^. This enzyme synthesizes O_2_^•−^ instead of NO^•[5,13,18]^. Monoamine oxidase-A (MAO-A) is also involved in the production of O_2_^•−^ and H_2_O_2_ in myocardial tissues^[19–22]^. Lipoxygenase synthesizes O_2_^•−^, H_2_O_2_, and fatty acid hydroperoxides^[12,23]^. Cyclooxygenase produces O_2_^•−^ and H_2_O_2_^[23]^.

## Cardiac ROS production during myocardial ischemia/reperfusion

In 1980, it was reported that hypoxic perfusion of the isolated rat heart for 20–80 min had no effect on malondialdehyde (MDA) levels in myocardial tissues^[24]^. However, reoxygenation for 20–80 min resulted in a 3-fold increase in MDA levels in the heart, particularly after 60–80 min of reoxygenation^[24]^. Hypoxia and reoxygenation led to a decrease in the levels of glutathione in myocardial tissues. When the isolated perfused rabbit heart was subjected to hypoxia (1 h) and reoxygenation^[25]^, and creatine kinase (CK) was used as a marker of cell injury, SOD had no significant effect on CK release, but adding catalase to the perfusion solution reduced CK release; the XO inhibitor allopurinol or the Fe^2+^ chelator deferoxamine also abolished reoxygenation-induced cardiac injury. Therefore, the authors concluded that reoxygenation cardiac injury was mediated *via* an increase in hydrogen peroxide production, which may then form hydroxyl radical in Fenton and Haber-Weiss reactions^[25]^. It should be noted that XO was not detected in the rabbit heart^[26]^, thereby it is unclear why allopurinol protected the rabbit heart. However, in another study, allopurinol (a 24-h oral pretreatment, 400 mg, then 50 mg/kg body weight intravenous bolus on occlusion) or SOD (starting with occlusion at a dose of 15 000 U/kg) decreased infarct size in dogs^[27]^, and thus the investigators concluded that XO was a source of ROS that injured the canine heart in I/R. N-tert-butyl-α-phenylnitrone (PBN) was used as a spin-trapping agent in a study performed with the isolated perfused rat heart^[28]^, in which, the isolated rat heart was subjected to global ischemia (15 min) and reperfusion (30 min); the free radical level was low during ischemia, but the amount of free radicals increased significantly during reperfusion, reaching a maximum 2 min after the restoration of coronary flow; although the amount of free radicals increased during anoxic reperfusion, their level peaked 10 min after the onset of reperfusion^[28]^; consequently, reperfusion causes the formation of the main oxygen free radicals. When the isolated perfused rabbit heart was subjected to ischemia (10 min) and reperfusion (10 s)^[10]^, 10-s reperfusion induced an increase in the amount of oxygen free radicals in myocardial tissues. In this case, the reperfusion was reported to trigger the formation of O_2_^•−^, ^•^OH, and R^•^ in myocardial tissues^[10–11]^.

When dogs underwent coronary artery occlusion (CAO, 15 min) and reperfusion (180 min)^[29]^, after intracoronary infusion of the spin trap PBN, electron paramagnetic resonance signals of oxygen- and carbon-centered radical adducts were detected in the coronary blood effluent during CAO and reperfusion; however, PBN adducts were not detected during ischemia but were found during reperfusion, and the concentration of PBN adducts in coronary effluent reached its maximum 3–5 min after the onset of reperfusion. When the isolated perfused rabbit heart was subjected to global ischemia (10 min) and reperfusion (60 s)^[30]^, the maximum free radical production occurred at 20 s of reperfusion and then slowly decreased^[30]^, but the high level of PBN adducts persisted through the first 10 min of reperfusion as seen in another study^[29]^. When dogs were subjected to CAO (15 min) and reperfusion (4 h)^[31]^, deferoxamine was infused intracoronary 2 min before reperfusion or 1 min after reperfusion; deferoxamine improved the recovery of contractile function, when it was administered before reperfusion; however, it showed no effect, when it was injected after the onset of reperfusion; when intracoronary infusion of the spin trap PBN was used to detect free radicals in coronary blood effluent, it was found that administration of deferoxamine before reperfusion reduced PBN adducts released from the heart during reperfusion; therefore, the investigators believed that this result indicated that iron-catalyzed free radical production was developed in the initial seconds of reperfusion and that these radicals could be responsible for reperfusion myocardial stunning^[31]^. In another study, the isolated perfused rat heart was subjected to global ischemia (15 min) and reperfusion (30 min)^[32]^, and the heart was perfused with a buffer containing PBN before ischemia (5 min) and during reperfusion. As a result, PBN adducts were found in the coronary effluent during reperfusion, and the amounts of free radicals reached a maximum 2 min after reperfusion and then rapidly decreased. A similar time course of the PBN adducts was also observed by other investigators^[28–29,31]^, in which the hearts were perfused with the perfusion solution containing catalase, SOD, or deferoxamine before ischemia and after ischemia with allopurinol administered orally; SOD or deferoxamine partially decreased but not abolished the increase of the PBN adduct levels. However, in another study, catalase and allopurinol completely abolished the increase in the concentration of PBN adducts^[32]^. These findings indicate that XO is involved in reperfusion free radical production in rat myocardium, and the isolated perfused rabbit heart underwent I/R. One study found that SOD or deferoxamine partially reduced but not abolished the increase of free radical production after reperfusion^[33]^, when the isolated perfused rat heart was subjected to 30 min of ischemia, followed by 30 min of reperfusion^[34]^. In this case, reperfusion triggered ^•^OH generation in myocardial tissues, the peak of ^•^OH production occurred at 1–2 min of reperfusion, and deferoxamine did not abolish ^•^OH formation; consequently, the Fenton reaction was not involved in ^•^OH generation. These data indicate that ^•^OH, O_2_^•−^, and H_2_O_2_ are formed in reperfusion of the heart. When the conscious dogs underwent CAO (15 min) and reperfusion (48 h), pretreatment with deferoxamine (500 mg) or N-2-mercaptopropionyl glycine (MPG, 100 mg/kg) improved the recovery contractile function of the heart in reperfusion^[35]^, and the authors claim that MPG is a ^•^OH scavenger^[35]^, while most investigators consider MPG not a selective ^•^OH scavenger. This study also found that both deferoxamine and MPG improved reperfusion myocardial contractility and that they almost completely abolished an increase in PBN adducts in reperfusion^[35]^. In one study, the isolated rat heart was subjected to global ischemia (5, 10, 20, 30, and60 min) followed by 10 min of reperfusion with the buffer containing 25 μmol/L β-carotene used for detection of singlet oxygen (^1^O_2_)^[36]^, in which the concentration of 5,8-endoperoxide in coronary effluent was used as an indicator of ^1^O_2_ production and reperfusion induced ^1^O_2_ generation; a maximal increase in ^1^O_2_ production was found after 60-min ischemia, and pretreatment with a ^1^O_2_ scavenger histidine reduced the 5,8-endoperoxide level in coronary effluent by 90%^[36]^. In another study, the isolated perfused rat heart was subjected to global ischemia (20 min) and reperfusion^[37]^, and ROS production was measured as 5,5-dimethyl-1-pyrroline-N-oxide (DMPO) adducts in the coronary effluent by using electron paramagnetic resonance spectroscopy; the peak of ROS generation was detected 2 min after the reperfusion; in addition, hypoxic (2% O_2_), normoxic (20% O_2_), and hyperoxic reperfusions (95% O_2_) were performed, and the maximal DMPO adduct level was measured in hypoxic reperfusion; it was found that hyperoxic reperfusion inhibited ROS production, and that the concentration of oxygen was not a limiting factor for ROS generation; instead, Tiron, an O_2_^•−^ scavenger, allopurinol, and diphenyleneiodonium, an NOX inhibitor, reduced the DMPO adduct level. Because Tiron exhibited a strong antioxidant effect, while allopurinol induced a weaker effect, the investigators concluded that there were different pathways of ROS production in the heart.

ROS formation was observed not only in cardiomyocytes at I/R but also in endothelial cells in anoxia/reoxygenation (A/R). In one study, bovine pulmonary artery endothelial cells were subjected to A/R^[38]^, in which A/R triggered an increase in the DMPO levels in cells, and allopurinol reduced but did not abolish ROS production, but SOD and catalase decreased free radical levels more effectively than allopurinol^[38]^. Free radical generation in human vascular endothelial cells subjected to A/R was detected by electron paramagnetic resonance measurements with the spin trap DMPO^[39]^, in which cells were subjected to anoxia (60 min) and reoxygenation (10 min); the results suggested that SOD, catalase, and oxypurinol, an XO inhibitor, completely abolished reoxygenation ROS formation and that deferoxamine reduced but not abolished free radical generation. These findings indicate that XO is the main source of free radicals in endothelial cells. However, there are other sources of ROS in endothelial cells. For example, one study reported that I/R of the isolated rat heart triggered the coronary artery dysfunction^[40]^ and decreased the response to acetylcholine (34% of preischemic value); treatment with tert-butyl hydroperoxide, a ROS donor, also induced coronary artery dysfunction, but the coronary artery response to glyceryl trinitrate did not alter. Therefore, the investigators concluded that I/R reduced endothelium-dependent vasodilatation of coronary arteries, but endothelium-independent vasodilatation did not alter. They also hypothesized that ROS was involved in reperfusion coronary artery dysfunction.

As mentioned above, we have presented data to elucidate that XO is one of the sources of free radicals in myocardial tissues. What are other sources of ROS that exist in cardiomyocytes? NOX has been found to be an important source of O_2_^•−^ in the heart^[41–42]^, while mitochondria also generate superoxide radicals^[9,43–46]^.

Hypoxia/reoxygenation induced transformation of NOS to uncoupled NOS that synthesized O_2_^•−^ instead of NO^•[18]^. Neutrophil invasion is involved in reperfusion cardiac injury^[47–48]^, and neutrophils can induce oxidative stress in myocardial tissues^[49]^. However, it is unclear whether ROS generated by neutrophils triggers reperfusion cardiac injury. It was reported that MAO was also involved in the I/R-induced ROS production in myocardial tissues^[21–22]^.

These findings indicate that ^1^O_2_, ^•^OH, O_2_^•−^, and H_2_O_2_ are formed in reperfusion of the heart. The peak of ROS generation occurs in the first 2 min of reperfusion. Thus, ROS in myocardial tissues are synthesized by XO, NOX, mitochondria, uncoupled NOS, lipoxygenase, and MAO.

## Are ROS responsible for myocardial ischemia/reperfusion injury?

**Figure 1:**
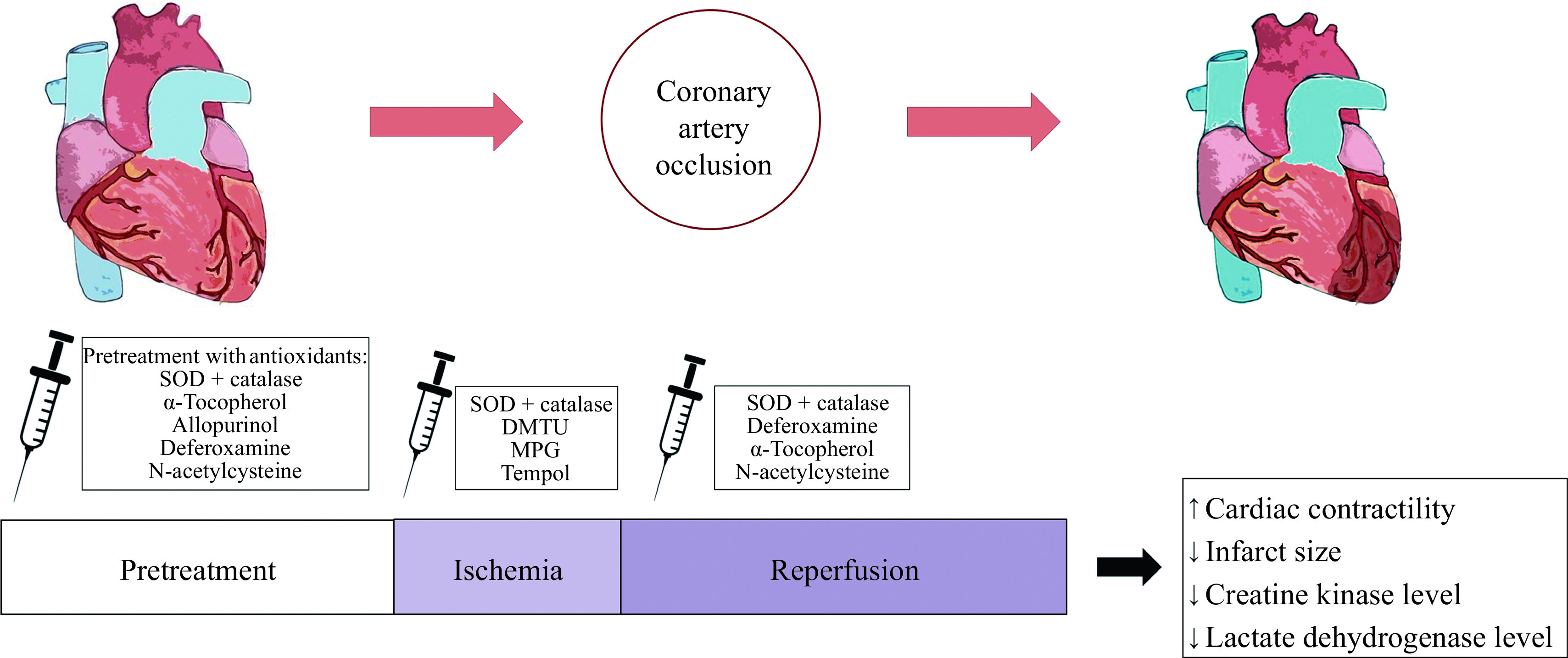
The antioxidants-triggered cardioprotection.

When the isolated perfused rabbit heart was subjected to hypoxic perfusion for 30 min^[50]^ and α-tocopherol was added to the perfusion solution, α-tocopherol improved cardiac contractility and reduced the CK and lactate dehydrogenase (LDH) levels in coronary effluent during hypoxia (***Fig. 1***)^[50]^. However, it is unclear whether these effects of α-tocopherol depend upon its antioxidant properties. When the isolated rat heart was subjected to hypoxic perfusion (60 min) and reoxygenation (20 min)^[51]^ and reduced glutathione, SOD, catalase, and α-tocopherol were added to the perfusion solution, all compounds decreased the CK levels in coronary effluent. Specifically, α-tocopherol exhibited the most potent cardioprotective effect and reoxygenation induced a rapid increase in MDA release from the heart. All compounds and mannitol, a quencher of ^•^OH, decreased MDA release, and both α-tocopherol and SOD exhibited the most potent antioxidant effect^[51]^. Therefore, free radicals can injure the heart in reoxygenation. Another study found that SOD reduced infarct size, while catalase had no effect on dogs with CAO and reperfusion^[52]^. When dogs received 400 mg of allopurinol orally one day before surgery, and at a dose of 25 mg/kg bolus intravenously immediately before occlusion, and repeated every 8 h^[53]^, the XO inhibitor allopurinol reduced the infarct size in dogs with CAO and reperfusion (24 h)^[53]^. When dogs underwent CAO (90 min) followed by reperfusion (6 h)^[54]^ and received allopurinol (25 mg/kg) 18 h before CAO and 50 mg/kg 5 min before CAO, allopurinol reduced the infarct size; in addition, pretreatment with SOD and catalase promoted improvement of cardiac contractility in reperfusion in dogs (***Fig. 1***)^[55]^. Another study reported that when dogs underwent CAO (90 min) and reperfusion (48 h)^[56]^, SOD reduced the infarct size. SOD and catalase also decreased the infarct size in pigs (***Fig. 1***)^[57]^, while MPG improved contractile function of the heart in dogs after CAO (15 min)^[58]^. When dogs underwent CAO (15 min) followed by 4 h of reperfusion, the ^•^OH scavenger dimethylthiourea (DMTU) at a dose of 500 mg/kg intravenously promoted the improvement of cardiac contractility during reperfusion^[59]^. In another study, dogs underwent a 15-min CAO, followed by 4 h of reperfusion^[60]^, allopurinol was administered at a dose of 50 mg/kg 48 h, 20 h, and 30 min before CAO, 10 mg/kg one minute before reperfusion, and 6.25 mg/(kg·h) during reperfusion. As a result, allopurinol improved cardiac contractility during reperfusion. When dogs were subjected to CAO(15 min) and reperfusion (3 h)^[61]^ and deferoxamine (500 mg intra-atrially) was infused 15 min prior to and throughout a 15-min of CAO, deferoxamine improved the recovery of contractile function during reperfusion (***Fig. 1***), and stimulated the recovery of ATP levels in the reperfused myocardium^[61]^. When dogs were subjected to a 90-min CAO and then reperfused for 6 h^[62]^ and received polyethylene glycol conjugated SOD (PEG-SOD, 1000 U/kg), the infarct size was reduced.

The cardioprotective effect of PEG-SOD was confirmed by other investigators^[63]^. For example, MPG [8 mg/(kg·h)] infusion prevented the development of reperfusion contractile dysfunction in dogs undergoing CAO (15 min) and reperfusion (4 h)^[63–64]^. MPG reduced the amount of PBN adducts in coronary blood effluent, and MPG (1.5 mmol/L) had no effect on XO activity^[64]^. MPG reacts slowly with H_2_O_2_, but the very small rate constant for this reaction suggests that it may not be significant at the micromolar concentrations of H_2_O_2,_ which is likely to be present *in vivo*^[64]^. In the radiolysis of a dilute (10 mmol/L) phosphate-buffered (pH 7.0) solution saturated with nitrous oxide produces ^•^OH, Bolli *et al* demonstrated that MPG was an excellent scavenger of ^•^OH. MPG, at concentrations up to 0.5 mmol/L, had no inhibitory effect on iron-induced lipid peroxidation in rat liver microsomes^[64]^. Consequently, MPG exhibits the properties of a selective ^•^OH scavenger *in vitro*. However, it is unknown at what dose that MPG exhibits the properties of a selective ^•^OH scavenger *in vivo*. In a study, rats underwent CAO (25 min) and reperfusion (2 h)^[65]^, tempol (100 mg/kg, a membrane-permeable O_2_^•−^ scavenger), was injected 5 min before reperfusion followed by an infusion of 30 mg/(kg·h) during reperfusion. A total dose of tempol was 160 mg/kg. As a result, tempol decreased the infarct size by 60% (***Fig. 1***)^[65]^. It should be noted that tempol is usually used at a dose of 30 mg/kg^[66]^. According to our own data, tempol at a dose of 30 mg/kg completely abolished the infarct-reducing effect of remote post-conditioning in rats (unpublished data). Therefore, the molecular mechanism of the infarct-reducing effect of tempol at a dose of 160 mg/kg remains unknown.

It was reported that the NOX inhibitor VAS2870 limited the infarct size by 15% in mice^[67]^, but the significance of this finding is questionable, because the effect was weak, and the group of animals consisted of only six mice. In one study, H9c2 cells were incubated for 10 min with PTD-copper-zinc SOD (PTD-CuZnSOD), where PTD is protein transduction domain^[68]^, and this enzyme penetrated the cells and prevented hypoxia/reoxygenation induced apoptosis of H9c2 cells. The investigators did not evaluate the survival of H9c2 cells, thereby it remains unknown whether SOD could increase the viability of these cells under hypoxia/reoxygenation. In another study, the isolated rat heart was subjected to global ischemia (30 min) and reperfusion (30 min)^[69]^, and a free radical donor photoactivated Rose Bengal aggravated I/R injury of the heart. In addition, SOD and catalase mitigated but did not abolish this injury, while histidine, an ^1^O_2_ scavenger, showed no effects. These findings indirectly demonstrate that free radicals (O_2_^•−^, ^•^OH) can damage the heart (***Fig. 1***), but ^1^O_2_ does not.

As mentioned above, we presented the results of studies using antioxidants. There is another approach using animals with altered gene expression. It has been demonstrated that glutathione peroxidase is the scavenger of H_2_O_2_ and phospholipid hydroperoxides^[70]^. In one study, when the isolated perfused hearts of mice with glutathione peroxidase overexpression were subjected to global ischemia(30 min) and reperfusion (20 min)^[71]^, transgenic mouse hearts demonstrated improved recovery of contractile function, compared with non-transgenic control mouse hearts; Furthermore, the infarct size and CK release were lower in transgenic murine hearts, compared with those of non-transgenic controls^[71]^. Consequently, glutathione peroxidase overexpression promoted an increase in cardiac tolerance to I/R. In another study, when the isolated hearts of mice with SOD overexpression were subjected to ischemia (6 min) and reperfusion (10 min)^[72]^, SOD overexpression promoted a significant increase in the rate of contraction by approximately 3%. It is surprising why such a weak effect was significant. When the isolated hearts of CuZnSOD-deficient mice were subjected to global ischemia (30 min) and reperfusion (120 min)^[73]^, *Sod1*^−/−^ knockout delayed the recovery of contractile function in reperfusion and increased reperfusion CK release.

It was reported that cardiomyocytes express three isoforms of NOX: NOX1, NOX2, and NOX4^[42]^. When mice with these genes knocked out and wild-type (WT) mice were subjected to CAO (30 min) and reperfusion (24 h), the infarct size was reduced by approximately 40% in *Nox1*^−/−^ mice and approximately 60% in *Nox2*^−/−^ mice, but the *Nox4* knockout showed no effect on the infarct size. Consequently, NOX1 and NOX2 are believed to be involved in reperfusion cardiac injury, but NOX4 does not participate in cardiac injury in I/R. When the isolated perfused hearts of WT mice and mice with altered expression of NOX4 were subjected to low-flow global ischemia (3% of baseline, 25 min) and reperfusion (60 min)^[74]^, the overexpression of NOX4 promoted the increase in the infarct size; however, NOX4 overexpression contributed to the improvement of contractile function of the heart in reperfusion and reduced lactate release and the creatine phosphate levels in myocardial tissues. It was surprising that NOX4 overexpression promoted oxidative stress and increased free radical production in myocardial tissues^[74]^. The contradictory nature of these data are probably due to the small sample size, and thus the significance of the data is questionable. When the isolated hearts of WT mice, *Sod1*^+/−^, and *Sod2* knockout mice were subjected to ischemia (30 min) and reperfusion (60 min)^[75]^, *Sod1*^+/−^ knockout reduced CuZnSOD by approximately 50% but had no effect on cardiac tolerance to I/R, while *Sod2* knockout aggravated I/R injury^[75]^. Consequently, CuZnSOD is not involved in the regulation of cardiac resistance to I/R, while MnSOD increases cardiac tolerance to I/R.

In summary, some studies have demonstrated the involvement of ROS in I/R cardiac injury^[50–57,62–66]^. Data from some studies are questionable^[67–68,74]^. The results of genetic studies seem to convincingly demonstrate the involvement of ROS in I/R cardiac injury. However, it should be noted that ROS can regulate transcription of genes^[76–78]^. For example, it was demonstrated that *Nox2*^−/−^ knockout altered the expression of some genes^[79]^. Therefore, the chronic reduction of ROS production or a chronic increase in ROS formation could change the expression of genes that regulate cardiac tolerance to I/R. Chronic administration of antioxidants could also alter the expression of genes. For example, α-tocopherol is itself a regulator of transcription of genes^[80]^.

## Can ROS be beneficial during myocardial ischemia/reperfusion?

**Figure 2:**
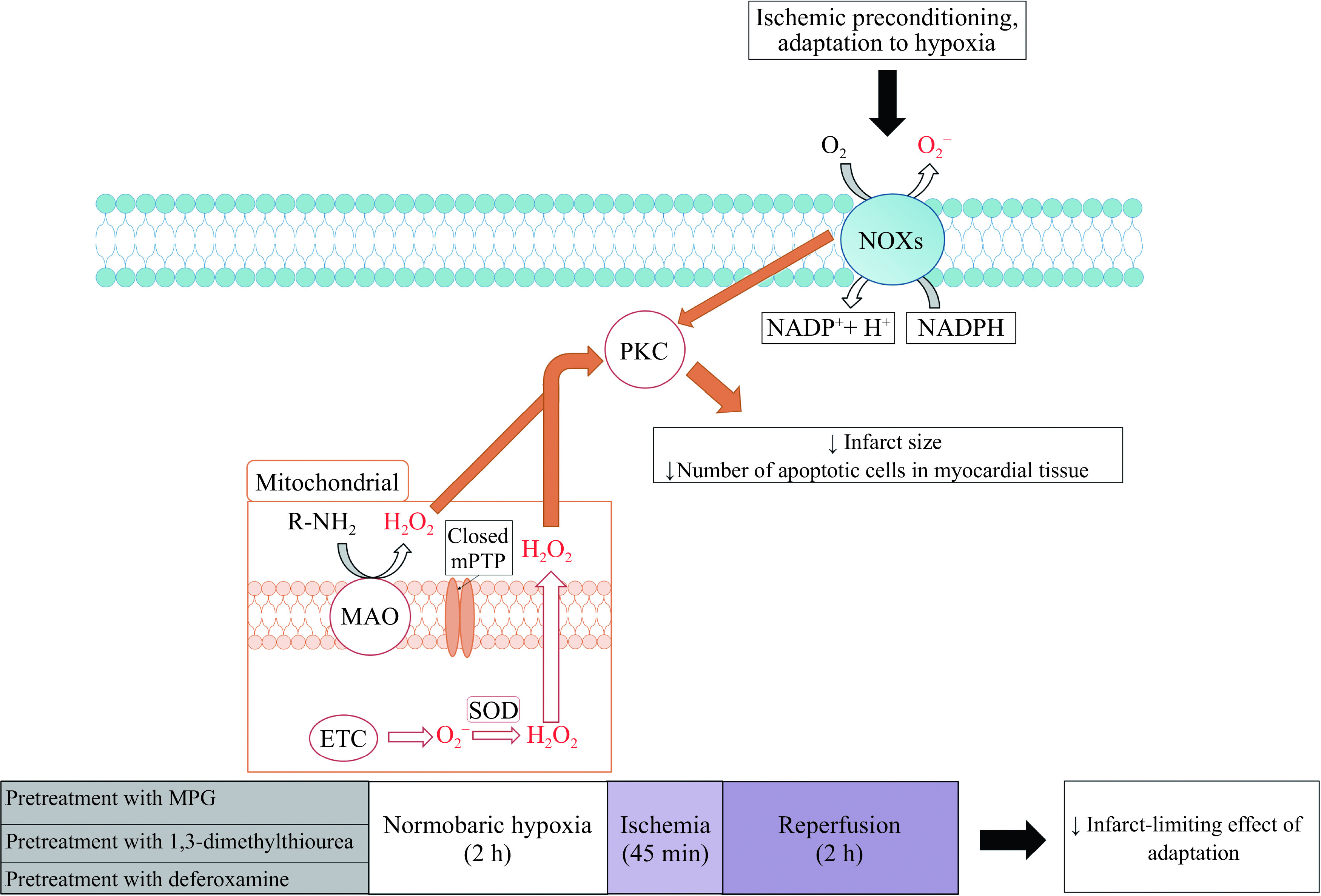
Signaling mechanism of reactive oxygen species triggering cardioprotection.

It was reported that MPG at a dose of 20 mg/kg intravenously had no effect on infarct size in animals with CAO and reperfusion^[4,81–82]^. We also found that MPG (20 mg/kg) had no influence on infarct size (unpublished data). However, this dose was completely enough to abolish the infarct-reducing effect of ischemic preconditioning^[4]^ and postconditioning^[81]^. These data indicate that free radicals protect the heart against I/R injury and mediate the infarct-reducing effect of preconditioning and postconditioning (***Fig. 2***). Ischemic preconditioning reduced infarct size in wild-type mice but had no effect on that of *Nox2* knockout mice^[82]^. Consequently, O_2_^•−^ synthesized by NOX2 promoted an increase in cardiac tolerance to I/R. When isolated hearts of wild-type and *Nox2*^−/−^ knockout mice were subjected to global ischemia (30 min) and reperfusion (120 min)^[79]^, *Nox2* knockout had no effect on the infarct size but abolished the infarct-reducing effect of ischemic preconditioning. These data indirectly confirmed our hypothesis that O_2_^•−^ synthesized by Nox2 contributed to an increase in cardiac tolerance to I/R (***Fig. 2***). However, it remains unknown why its cardioprotective effect was observed only in ischemic preconditioning. When wild-type (WT) mice and *Nox2*, *Nox4*, *Nox2*/*Nox4* double knockout mice underwent CAO (30 min) and reperfusion (24 h)^[83]^, *Nox2* or *Nox4* knockout decreased the infarct size and reduced a number of apoptotic cells in myocardial tissues; however, *Nox2*/*Nox4* double knockout induced an increase in the infarct size and the number of apoptotic cells in myocardial tissues^[83]^. It remains unclear why in one case, NOX2 and NOX4 were involved in I/R injury of the heart, while in another case they prevented I/R injury? When WT and *Nox2* knockout mice underwent CAO (30 min) and reperfusion (24 h)^[84]^, ischemic preconditioning reduced the infarct size by about 60% in both WT and *Nox2* knockout mice, but the infarct-reducing effect of late preconditioning was demonstrated only in WT mice. Consequently, the authors concluded that O_2_^•−^ synthesized by NOX2 was involved in a delayed increase in cardiac tolerance to I/R^[84]^. The mechanism of the cardioprotective effect of O_2_^•−^ remains unclear. It has been demonstrated that ROS activates protein kinase C (PKC)^[5]^. This enzyme mediated cardioprotective effect of ischemic preconditioning^[4]^. It is possible that O_2_^•−^ synthesized by NOX2 activates PKC that increased cardiac tolerance to I/R (***Fig. 2***). MPG, an ^•^OH scavenger, 1,3-dimethylthiourea, an ^•^OH scavenger, and deferoxamine had no effect on infarct size in non-adapted rats^[85]^. However, these antioxidants abolished the infarct-reducing effect of hypoxic preconditioning^[85]^. These findings indirectly demonstrate that ^•^OH could also be an intracellular messenger mediating the increase in cardiac tolerance to I/R.

In summary, these findings have demonstrated that O_2_^•−^ synthesized by NOX2 is involved in the cardioprotective effect of ischemic preconditioning (***Fig. 2***). However, these data were obtained from studies performed in *Nox2* knockout mice. As noted above, knockout itself results in alteration of the expression of genes. Although the results of studies using ^•^OH scavenger MPG are more convincing, many researchers doubt its selectivity with respect to ^•^OH.

## Are antioxidants cardioprotective?

When conscious dogs underwent CAO (3 h), followed by reperfusion (24 h)^[86]^, and SOD (5 mg/kg) or catalase (5 mg/kg) was infused 15 min before reperfusion and lasted for 45 min during reperfusion, SOD or catalase failed to decrease the infarct size^[86]^. It should be noted that the duration of CAO in most studies is 30–60 min. It is possible that SOD or catalase reduces infarct size, if the duration of CAO is less than 3 h. However, Uraizee *et al* could not find the infarct-reducing effect of SOD in dogs with a 40-min CAO and reperfusion^[87]^. When isolated rat hearts were subjected to anoxic perfusion (30 or 60 min) followed by a 20-min reoxygenation (oxygen paradox)^[88]^ and the heart injury was evaluated by the CK level in coronary effluent, the oxygen paradox triggered cardiac injury. Allopurinol or the ^•^OH scavenger DMTU did not abolish cardiac injury, because allopurinol caused a significant reduction in MDA release but not CK release from oxygen paradox-injured hearts^[88]^. These findings indicate that XO and ^•^OH are not involved in reoxygenation cardiac injury. Wang *et al* could not confirm the infarct-reducing effect of SOD in dogs with CAO and reperfusion^[67]^. It was reported that SOD had no effect on infarct size in dogs with CAO (40 min) and reperfusion (4 days)^[87]^. Allopurinol did not reduce infarct size in rabbits with CAO (45 min) and reperfusion (3 h) as myocardial tissues of these animals do not contain XO^[26]^. It was reported that *Nox4* knockout had no effect on infarct size in mice with CAO (30 min) and reperfusion (24 h)^[42]^, and ^1^O_2_ did not damage the heart^[69]^. CuZnSOD was reported to not be involved in the regulation of cardiac resistance to I/R^[75]^. We also found that tempol (an O_2_^•−^ scavenger) and mannitol (an ^•^OH scavenger) had no effect on the infarct size in rats with CAO (45 min) and reperfusion (120 min) (unpublished data). In addition, MPG, 1,3-dimethylthiourea, an^ •^OH scavenger, and deferoxamine also had no influence on the infarct size in non-adapted rats^[85]^.

## Update on the use of antioxidants in clinical cardiological practice

In 1997, the results of a clinical study of antioxidants emoxipin and hystochrom were reported^[89]^, in which emoxipin was administered intravenously at a dose of 3 mg/kg; hystochrom was injected intravenously at a dose of 1 mg/kg before coronary artery bypass grafting (CABG), which was performed using cardioplegic cardiac arrest, the essentially global I/R of the heart. The control group included patients who did not receive these antioxidants. Both of these antioxidants contributed to a decrease in the serum CK-MB level during the postoperative period. Hystochrom had a more pronounced cardioprotective effect. The state of lipid peroxidation was evaluated by the serum MDA level. Both of these antioxidants reduced the MDA levels, but hystochrom had a more pronounced effect^[89]^. Emoxipin contributed to a decrease in the plasma conjugated dienes' level among patients with CABG, and a decrease in the incidence of arrhythmias during the postoperative period and defibrillation threshold^[90]^. In a study performed among patients with AMI, who received thrombolytic therapy, intravenous administration of hystochrom at a dose of 100 mg resulted in a decrease in the plasma CK level^[91]^.

The aforementioned clinical studies indicate that antioxidant emoxipin and hystochrom have a cardioprotective effect, when the heart is exposed to I/R. N-acetylcysteine (NAC, 100 mg/kg intravenously) was administered to patients with AMI and recanalization of the infarct-related coronary artery in the ISLAND (Infarct SIZE Limitation: Acute NAC Defense) trial, performed in 1996^[92]^. The investigators demonstrated an improvement in left ventricular contractility of patients treated with NАС, compared with the control group (streptokinase) (***Fig. 1***). In 2006, other results of this trial were reported^[93]^. When NAC was infused at a dose of 15 g, this antioxidant did not affect the plasma CK-MB levels, but reduced the MDA levels and promoted the increase of left ventricular ejection fraction. A randomized, double-blind, placebo-controlled NACIAM trial (NAC) with a nitrate therapy in patients undergoing primary percutaneous coronary intervention (PCI) for ST-segment elevation myocardial infarction reduced myocardial infarct size, and this trial included patients with AMI and PCI, who were infused with NAC at a dose of 29 g for 2 days^[94]^. Magnetic resonance imaging (MRI) evaluation revealed that NAC resulted in an infarct size reduction by 33% (***Fig. 1***), but it did not affect the plasma CK level. Thus, the result was inconsistent: the MRI data indicated that NAC reduced the infarct size, but the biochemical CK results indicated no benefit on the infarct size. In the following study, myocardial infarction patients with ST-segment elevation and PCI were included^[95]^ with NAC administered intracoronarily. The trial included patients (*n* = 58) with CABG who received NAC (600 mg per os) before CABG^[96]^. The results showed that NAC did not affect the CK-MB level but contributed to a 32% decrease in the serum troponin T level and that NAC promoted a decrease in the serum lactate and MDA level during reperfusion but did not affect troponin I. Therefore, NAC exhibited an antioxidant effect but did not prevent reperfusion necrosis of cardiomyocytes.

A study performed in 2006^[97]^ included patients with AMI who underwent primary stenting of the infarct-related coronary artery. This study indicated that administration of antioxidant edaravone at a dose of 30 mg contributed to a decrease in the serum peak CK and CK-MB levels. After the use of edaravone, a decrease in the incidence of reperfusion arrhythmias was also detected^[97]^, suggesting that edaravone had the cardioprotective effect in patients with AMI.

In 2012, the results of the clinical trial of the iron ion chelator deferoxamine were reported^[98]^. The study was performed among patients with AMI and PCI, and deferoxamine was infused intravenously at a dose of 500 mg. This antioxidant did not affect the plasma CK and troponin I levels; moreover, deferoxamine had no effect on the infarct size according to MRI. However, it was also noted that patients receiving deferoxamine had a 75% decrease in plasma iron levels in comparison with the control group. Deferoxamine was found to be able to lower plasma levels of F2-isoprostane, which is an indicator of ROS *in vivo*. These studies indicate that deferoxamine has an antioxidant effect but not an infarct-reducing effect.

In 1994, Coghlan *et al* reported that pretreatment with allopurinol decreased the use of inotropic support, increased the cardiac index, and reduced the serum MDA level in patients with CABG^[99]^. Pretreatment with allopurinol decreased the serum MDA levels and the serum CK-MB levels during reperfusion in patients with CABG^[100]^. In a study included 140 patients with STEMI, the participants received 400 mg of allopurinol or placebo before using streptokinase^[101]^. Those treated with allopurinol had a 47% lower peak CK level (*P* = 0.003), a 27% lower peak CK-MB (*P* = 0.005) and a 46% lower peak troponin I level (*P* < 0.001), compared with the control group. The authors believed that allopurinol treatment resulted in a pronounced infarct-reducing effect. The results of this study are surprising, because XO is absent in human myocardium. Apparently, the cardioprotective effect of allopurinol is not associated with the decreased O_2_^•−^ production in cardiomyocytes. Why allopurinol decreases MDA levels remains a mystery. It is possible that the protective effect of allopurinol is a result of myeloperoxidase inhibition in neutrophils of the reperfused heart^[102]^.

Unfortunately, most of the aforementioned clinical trials were not blinded and placebo-controlled and were performed in small groups of patients, so it is difficult to conclusively report about the cardioprotective effect of antioxidants. In a blind, placebo-controlled study utilizing NAC, conflicting data were obtained. The results of the above-cited clinical studies indicate that the following antioxidants have cardioprotective properties: emoxipin, hystochrom, edaravone, and allopurinol.

## Conclusions

In 1990, J.M. Downey wrote: "After more than a decade of research on the free radical hypothesis, there is still little agreement on the role free radicals actually play in the reperfused myocardium"^[103]^. There is no doubt that reperfusion triggers an increase in ROS generation. However, many questions remain unanswered, as it was in 1990.

Some authors have demonstrated that free radicals are involved in I/R injury of the heart, because antioxidants increased cardiac tolerance to I/R. It should be noted that selectivity of some antioxidants to free radicals remains unclear before. The XO inhibitor allopurinol inhibits not only XO but also myeloperoxidase of neutrophils involved in reperfusion cardiac injury^[102]^. MPG at a dose of 70 mg/kg can interact not only with ^•^OH but also eliminate peroxynitrite^[104]^. Investigators who found the cardioprotective effect of antioxidants usually used high doses of these compounds, thereby it is highly likely that their cardioprotective effect is independent of the elimination of free radicals. The main drawback of knockout mice studies is that the knockout or the overexpression of genes induces alterations in ROS levels of the cell that triggers alterations in the expression of other genes.

There are a number of studies that failed to detect the infarct-reducing effect of antioxidants; as a rule, these researchers used small doses of antioxidants in studies performed with non-adapted animals. It was demonstrated that MPG or *Nox2* knockout abolished the infarct-limiting effect of ischemic preconditioning. Therefore, if there were ROS that protected the heart against I/R injury, it could be ^•^OH or O_2_^•−^.

It is currently unclear which free radicals damage the heart and which protect the heart against I/R. It is possible that a free radical plays the roles of both damaging and protecting the heart, depending upon free radical concentration in the cell. We proposed that a small increase (+15% to +25%) in serum MDA or 4-hydroxinonenal levels in patients with AMI was an indicator of response to stress as an increase in the cortisol levels. In contrast, a big increase (+100%) in serum MDA or 4-hydroxinonenal levels could be an indicator of organ injury. The boundary between injury and the adaptive response has not yet been defined. H. Selye (1952)^[105]^ demonstrated that stress was a normal, protective response of the body to an excessive stimulus. They found that the response was not developed in hypophysectomized rats, subsequently casuing them death when exposed to a strong stimulus, but it did not affect the survival of rats with preserved pituitary glands. Perhaps moderate oxidative stress can also play a protective role.

In our opinion, studies in the isolated heart using the low concentrations of the selective free radical scavengers with simultaneous detection of ROS production and cardiac injury are needed to resolve the issue of the role of ROS in reperfusion injury of the heart. A strong argument for the involvement of oxygen free radicals in the cardioprotection would be a study in which an oxygen free radical donor increased cardiac tolerance to I/R. It was reported that H_2_O_2_ (25 and 500 µmol/L) reduced hypoxia/reoxygenation-induced Ca^2+^ overload of isolated cardiomyocytes^[106]^. It was demonstrated that H_2_O_2_ (5 µmol/L) activated PKCε, induced mitochondrial ATP sensitive K^+^ channel (mitoK_ATP_ channel) opening, and increased the viability of cardiomyocytes of isolated chick embryos in hypoxia/reoxygenation^[107]^. It should be noted that PKCε and mitoK_ATP_ channel participate in the infarct-reducing effect of ischemic preconditioning^[4]^. It is unclear these positive effects of H_2_O_2_ exhibit themselves as H_2_O_2_ or ^•^OH that formed in the Fenton reaction. Consequently, there is evidence that ROS protect the heart against the I/R injury.
